# Interformat Reliability of Web-Based Parent-Rated Questionnaires for Assessing Neurodevelopmental Disorders Among Preschoolers: Cross-sectional Community Study

**DOI:** 10.2196/20172

**Published:** 2021-02-04

**Authors:** Masanori Tanaka, Manabu Saito, Michio Takahashi, Masaki Adachi, Kazuhiko Nakamura

**Affiliations:** 1 Faculty of Business Administration Hokkai-Gakuen University Sapporo, Hokkaido Japan; 2 Department of Neuropsychiatry Graduate School of Medicine Hirosaki University Hirosaki, Aomori Japan; 3 Graduate School of Health Sciences Hirosaki University Hirosaki, Aomori Japan; 4 Research Center for Child Mental Development Graduate School of Medicine Hirosaki University Hirosaki, Aomori Japan

**Keywords:** neurodevelopmental disorders, web-based questionnaire, preschoolers, parents, interformat reliability

## Abstract

**Background:**

Early detection and intervention for neurodevelopmental disorders are effective. Several types of paper questionnaires have been developed to assess these conditions in early childhood; however, the psychometric equivalence between the web-based and the paper versions of these questionnaires is unknown.

**Objective:**

This study examined the interformat reliability of the web-based parent-rated version of the Autism Spectrum Screening Questionnaire (ASSQ), Attention-Deficit/Hyperactivity Disorder Rating Scale (ADHD-RS), Developmental Coordination Disorder Questionnaire 2007 (DCDQ), and Strengths and Difficulties Questionnaire (SDQ) among Japanese preschoolers in a community developmental health check-up setting.

**Methods:**

A set of paper-based questionnaires were distributed for voluntary completion to parents of children aged 5 years. The package of the paper format questionnaires included the ASSQ, ADHD-RS, DCDQ, parent-reported SDQ (P-SDQ), and several additional demographic questions. Responses were received from 508 parents of children who agreed to participate in the study. After 3 months, 300 parents, who were among the initial responders, were randomly selected and asked to complete the web-based versions of these questionnaires. A total of 140 parents replied to the web-based format and were included as a final sample in this study.

**Results:**

We obtained the McDonald ω coefficients for both the web-based and paper formats of the ASSQ (web-based: ω=.90; paper: ω=.86), ADHD-RS total and subscales (web-based: ω=.88-.94; paper: ω=.87-.93), DCDQ total and subscales (web-based: ω=.82-.94; paper: ω=.74-.92), and P-SDQ total and subscales (web-based: ω=.55-.81; paper: ω=.52-.80). The intraclass correlation coefficients between the web-based and paper formats were all significant at the 99.9% confidence level: ASSQ (r=0.66, *P*<.001); ADHD-RS total and subscales (*r*=0.66-0.74, *P*<.001); DCDQ total and subscales (*r*=0.66-0.71, *P*<.001); P-SDQ Total Difficulties and subscales (*r*=0.55-0.73, *P*<.001). There were no significant differences between the web-based and paper formats for total mean score of the ASSQ (*P*=.76), total (*P*=.12) and subscale (*P*=.11-.47) mean scores of DCDQ, and the P-SDQ Total Difficulties mean score (*P*=.20) and mean subscale scores (*P*=.28-.79). Although significant differences were found between the web-based and paper formats for mean ADHD-RS scores (total: *t*_132_=2.83, *P*=.005; Inattention subscale: *t*_133_=2.15, *P*=.03; Hyperactivity/Impulsivity subscale: *t*_133_=3.21, *P*=.002), the effect sizes were small (Cohen *d*=0.18-0.22).

**Conclusions:**

These results suggest that the web-based versions of the ASSQ, ADHD-RS, DCDQ, and P-SDQ were equivalent, with the same level of internal consistency and intrarater reliability as the paper versions, indicating the applicability of the web-based versions of these questionnaires for assessing neurodevelopmental disorders.

## Introduction

In the American Psychiatric Association's Diagnostic and Statistical Manual for Mental Disorders, Fifth Edition (DSM-5), neurodevelopmental disorders are identified in the early developmental stages and are characterized by developmental deficits that lead to impairments in personal, social, academic, and vocational functioning [1]. Representative examples of such disorders include autism spectrum disorder, attention-deficit/hyperactivity disorder, and developmental coordination disorder. The core characteristics of autism spectrum disorder include 2 main dimensions—social communication and restricted, repetitive sensory–motor behaviors—that are irrespective of culture, race, ethnicity, or socioeconomic group [2]. Estimates of the total-population prevalence of autism spectrum disorder range from 2.2% to 3.2% [3,4]. The hallmarks of attention-deficit/hyperactivity disorder are developmentally impaired attention, motor hyperactivity, impulsivity, and the difficulties associated with them [5]. A recent meta-analysis [6] revealed that the estimated prevalence of children with attention-deficit/hyperactivity disorder is 3.4% in the general population. Developmental coordination disorder is characterized by marked impairment in the acquisition and execution of motor skills. This impairment significantly and sustainably interferes with activities of daily living, including academic achievement [1]. A recent review [7] reported that prevalence estimates for developmental coordination disorder among children range from 2% to 20%, with 5% to 6% being the most commonly reported prevalence rate.

It is known that children with these conditions not only have various secondary mental health problems [8-10] but also experience maladjustment in adulthood [11-13]. Since a number of studies [7,14,15] have reported that early detection and intervention for neurodevelopmental disorders is effective, it is necessary to develop useful screening tools for assessing these conditions in early childhood.

Several questionnaires have been developed to assess a variety of neurodevelopmental disorders. The Autism Spectrum Screening Questionnaire (ASSQ) was developed to screen for autism spectrum disorder in school-age children based on their parents’ or primary caregivers’ ratings [16] and has been shown to be highly accurate in screening for autism spectrum disorder [17]. Additional research has confirmed that the ASSQ has good reliability and validity and can be applied to preschool-age children, as well [18,19]. The ADHD-Rating Scale (ADHD-RS) is one of the most widely used questionnaires developed to assess ADHD symptoms in children age 5 to 18 years [20]. There are 2 versions of the ADHD-RS: the home form is rated by parents or primary caregivers of the children, and the school form is rated by teachers. It has been demonstrated that the ADHD-RS has good reliability and validity in preschool children [21]. Moreover, previous research has confirmed that the ADHD-RS shows higher sensitivity and specificity in parent ratings than those in teacher ratings among preschoolers [22]. The Developmental Coordination Disorder Questionnaire 2007 (DCDQ) was developed to identify children age 5 to 15 years who are at risk for developmental coordination disorder, based on parents’ ratings [23]. It has been found that the DCDQ has good psychometric properties and has been recommended for use in clinical practice as supplemental information for the diagnosis of children with developmental coordination disorder [7]. It is known that children with neurodevelopmental disorders have behavioral and emotional difficulties. The Strengths and Difficulties Questionnaire (SDQ) is a brief behavioral screening questionnaire about externalizing and internalizing problems in children [24]. It has been reported that using parent ratings for the SDQ has satisfactory reliability and validity in a community sample of 5- to 15-year-old children [25].

These questionnaires have been developed as paper-and-pencil type questionnaires and can be useful in individual clinical settings; however, to screen large populations in a local community for early detection of neurodevelopmental disorders, web-based versions are more efficient than the paper-and-pencil version because of the significant time and effort required to distribute and collect paper-and-pencil questionnaires. Not only can costs be saved, but also there are additional advantages; for example, we can automate the process of manual data entry after completing data collection [26]. In addition, using web-based questionnaires to collect data generally improves the quality of the data because the validation checks can incorporate prompts that alert respondents if they enter incorrect or incomplete answers [27]. Furthermore, web-based instruments expand the reach of assessments, which is particularly important under pandemic conditions when it is difficult or impossible to administer in-person assessments [28,29].

On one hand, several studies [30-32] have reported psychometric equivalence between web-based and paper-and-pencil versions of the questionnaires used to assess various psychological disorders; on the other hand, some studies have revealed psychometrically significant differences between the 2 formats [26,33,34]. Therefore, it is necessary to evaluate the comparability of web-based and paper-and-pencil versions of the questionnaires [35]. In particular, high interformat reliability, meaning the level of equality between different delivery formats, indicates that the psychometric properties of the instrument are independent of the delivery format [36]. However, to our knowledge, no studies have assessed the interformat reliability of web-based questionnaires that aim to assess neurodevelopmental disorders.

We aimed to examine the interformat reliability of the web-based versions of the ASSQ, ADHD-RS, DCDQ, and SDQ. Based on previous work [36], we confirmed interformat reliability from the following 3 perspectives. First, we verified the internal consistency of the web-based and paper-and-pencil formats of each questionnaire. Second, we examined the intraclass correlations between the 2 formats of each questionnaire to test intrarater reliability. Third, we investigated the mean score differences between the 2 formats of each questionnaire to confirm equivalence in quality.

## Methods

### Participants

This study was conducted as part of the Hirosaki Five-Year-Old Children Developmental Health Check-up Study (HFC Study), a large community-based cohort study initiated in 2013 that examined the impact of children's neurodevelopmental disorders and lifestyle habits on their adaptation and emotional and behavioral problems at age 5 years. Located in Aomori Prefecture in the northeastern part of Japan, Hirosaki City has approximately 175,000 residents, 1 university, and several colleges, and its main industry is agriculture.

Participants in this study were recruited in July 2018. The local government of Hirosaki City distributed a set of paper-based questionnaires for voluntary completion to the parents of 620 5-year-old children in the city via the municipal health center. The package included the ASSQ, ADHD-RS, DCDQ, parent-reported SDQ (P-SDQ), and demographic questions. Responses were received from 508 parents who agreed to participate in the study. After 3 months, 300 of the 508 respondents were randomly selected and informed of the objective of this study. The individuals who gave their written consent to participate were asked to complete web-based versions of these questionnaires. Participants were given an ID and password to complete the web-based survey on their own computers. There were no restrictions on the type of computer (eg, personal computer, tablet, or smartphone) that they could use to complete the survey. A total of 140 parents replied to the web-based format and were included in the final sample in the present study. Table 1 shows the demographic characteristics of this sample; Multimedia Appendix 1 contains the characteristics of the 368 people who did not respond to the web-based survey.

**Table 1:** Participants’ demographic characteristics.

Characteristics		Value (N=140), n (%)
**Children’s gender**		
	Boy	78 (55.7)
	Girl	62 (44.3)
**Children’s age (months)**		
	60	3 (2.1)
	61	25 (17.9)
	62	21 (15.0)
	63	25 (17.9)
	64	19 (13.6)
	65	32 (22.9)
	66	15 (10.7)
**Respondent**		
	Mother	127 (90.7)
	Father	13 (9.3)
**Childcare during daytime**		
	Nursery school	115 (82.1)
	Kindergarten	24 (17.1)
	Mother	1 (0.7)
**Household income (JPY^a^)**		
	<2 million	10 (7.1)
	2-4 million	44 (31.4)
	4-7 million	56 (40.0)
	7-10 million	18 (12.9)
	>10 million	7 (5.0)
	Don’t know	5 (3.6)

^a^JPY: Japanese Yen; an approximate exchange rate of US $1= 103.80 JPY.

### Measures

#### ASSQ

The ASSQ has 27 items that assess autistic features such as social interaction and communication problems, behaviors that are restrictive and repetitive, motor clumsiness, and other associated symptoms, including motor and vocal tics [16,17]. The items are rated on a 3-point scale ranging from 0 (not true) to 2 (true). A higher ASSQ score indicates more severe autistic problems. The total possible score of the ASSQ ranges from 0 to 54. In this study, we used the Japanese version of the ASSQ. A previous study [19] revealed that the ASSQ had good reliability (autism spectrum disorder clinical group: Cronbach α=.88; community group: Cronbach α=.87) and validity as a screening instrument for use with preschoolers in Japanese community settings.

#### ADHD-RS

The ADHD-RS includes 18 items to measure 2 features of attention-deficit/hyperactivity disorder: Inattention (9-item subscale) and Hyperactivity/Impulsivity (9-item subscale) [20]. It is evaluated on a 4-point scale ranging from 0 (not at all or rarely) to 3 (very often). Higher scores on the ADHD-RS indicate more severe attention-deficit/hyperactivity disorder problems, with total scores ranging from 0 to 54. This study used the Japanese version of the ADHD-RS home form [37]. A previous study [22] revealed that this version of the ADHD-RS had sufficient reliability (Inattention subscale: Cronbach α=.88, Hyperactivity/Impulsivity subscale: Cronbach α=.85) and validity to screen for children potentially living with attention-deficit/hyperactivity disorder in a community setting.

#### DCDQ

The DCDQ consists of 15 items organized into 3 subscales—Control During Movements (6 items), Fine Motor and Handwriting (4 items), and General Coordination (5 items) [23]. Parents or primary caregivers were asked to evaluate the degree of motor coordination in their children compared to that of other children of the same age on a 5-point scale ranging from 1 (not at all like your child) to 5 (extremely like your child). Lower scores indicate severe developmental coordination disorder symptoms. The total possible score ranges from 15 to 75. This study used the Japanese version of the DCDQ, which has sufficient criterion validity, fit indices, and internal consistency (Total: Cronbach α=.93; Control During Movements subscale: Cronbach α=.91; Fine Motor and Handwriting: Cronbach α=.91; General Coordination: Cronbach α=.81) when used with preschool- and school-age children [38].

#### P-SDQ

The P-SDQ includes 25 items that assess children’s strengths and difficulties on 5 different subscales (each comprising 5 items): Emotional Symptoms, Conduct Problems, Hyperactivity/Inattention, Peer Relationship Problems, and Prosocial Behavior [24,25]. Parents or principal caregivers rated the items on a 3-point scale ranging from 0 (not true) to 2 (certainly true). The score for each subscale is calculated by summing the scores of 5 items, ranging from 0 to 10. The Total Difficulties score is calculated by summing the 4 difficulty subscale scores, ranging from 0 to 40. Higher scores on the 4 difficulty subscales as well as the Total Difficulties score indicate more severe emotional and behavioral deficits. Meanwhile, a higher score on the Prosocial Behavior subscale represents a more positive aspect of prosocial behavior. In this study, we used the P-SDQ, which showed favorable psychometric properties in Japanese community-based samples (Total Difficulties: Cronbach α =.77; Emotional Symptoms: Cronbach α=.61; Conduct Problems: Cronbach α=.52; Hyperactivity/Inattention: Cronbach α=.75; Peer Relationship Problems: Cronbach α=.52; Prosocial Behavior: Cronbach α=.69) [39].

### Statistical Analysis

To test internal consistency, we calculated McDonald ω coefficients for the total and subscale scores of each measure, based on a previous study’s recommendation [40], for both web-based and paper formats. We also calculated intraclass correlation coefficients between the web-based and paper formats to evaluate the interformat reliability. Paired 2-tailed *t* tests were performed to evaluate mean score differences between the web-based and paper formats to examine equivalence in quality. A *P* value <.05 was statistically significant. Analyses were performed using SPSS software (version 25.0; IBM Corp) and R (version 4.0.3; R Foundation for Statistical Computing).

### Ethics

The research was performed in accordance with the ethical guidelines of the Declaration of Helsinki. The protocol of this study was approved by the Committee on Medical Ethics of Hirosaki University (IRB 2018-168). To protect personal data, we adhered to the city’s and the committee’s information security policies.

## Results

### Internal Consistency

Table 2 shows the McDonald ω coefficients for both formats of the questionnaires.

**Table 2:** McDonald ω coefficients for the web-based and paper versions of the ASSQ, ADHD-RS, DCDQ, and P-SDQ (N=140).

Scale and subscales		McDonald ω	
		Web-based	Paper
Autism Spectrum Screening Questionnaire		.90	.86^a^
**Attention-Deficit/Hyperactivity Disorder Rating Scale**			
	Total	.94	.93^b^
	Inattention	.90	.88^c^
	Hyperactivity/Impulsivity	.88	.87^c^
**Developmental Coordination Disorder Questionnaire**			
	Total	.94	.92^a^
	Control During Movement	.87	.86^a^
	Fine Motor/Handwriting	.88	.91^d^
	General Coordination	.82	.74
**Parent-rated Strength and Difficulties Questionnaire**			
	Total Difficulties	.81	.78
	Emotional Symptoms	.64	.70
	Conduct Problems	.55	.50
	Hyperactivity/Inattention	.78	.79
	Peer Relationship Problems	.57	.52
	Prosocial Behavior	.76	.80

^a^Calculated for 137 participants because of missing data.

^b^Calculated for 133 participants because of missing data.

^c^Calculated for 134 participants because of missing data.

^d^Calculated for 139 participants because of missing data.

Based on a previous study [41], an internal consistency coefficient below .70 is considered unacceptable, a coefficient from .70 to .79 is considered fair, a coefficient from .80-.89 is considered good, and a coefficient of .90 or above is considered excellent. The McDonald ω coefficients for both the web-based and paper formats of the ASSQ ranged from .86 to .90, indicating good to excellent internal consistency. The McDonald ω coefficients for both the web-based and paper formats of the overall ADHD-RS and its subscales ranged from .87 to .94, also indicating good to excellent internal consistency. Meanwhile, those for both the web-based and paper formats of the overall DCDQ and its subscales ranged from .74 to .94, indicating fair to excellent internal consistency. The McDonald ω coefficients for the web-based and paper formats of the Total Difficulties subscale and the subscales of the P-SDQ ranged from .52 to .81. Notably, the McDonald ω coefficients for both the web-based and paper versions of the Peer Relationship Problems and Conduct Problems subscales and the web-based version of the Emotional Symptoms subscale were all unacceptable [41], with coefficients ranging from .51 to .66.

### Intraclass Correlation Coefficients

Table 3 presents the intraclass correlation coefficients between each format for ASSQ, ADHD-RS, DCDQ, and P-SDQ.

**Table 3:** Intraclass correlation coefficients between the web-based and paper formats of the ASSQ, ADHD-RS, DCDQ, and P-SDQ (N=140).

Scale and subscales		Intraclass correlation^a^ (95% CI)
Autism Spectrum Screening Questionnaire		0.66^b^ (0.56-0.75)
**Attention-Deficit/Hyperactivity Disorder Rating Scale**		
	Total	0.72^c^ (0.62-0.79)
	Inattention	0.66^d^ (0.55-0.74)
	Hyperactivity/Impulsivity	0.74^d^ (0.65-0.80)
**Developmental Coordination Disorder Questionnaire**		
	Total	0.71^b^ (0.61-0.78)
	Control During Movement	0.71^b^ (0.62-0.79)
	Fine Motor/Handwriting	0.66^e^ (0.55-0.74)
	General Coordination	0.66 (0.56-0.75)
**Parent-rated Strength and Difficulties Questionnaire**		
	Total Difficulties	0.73 (0.65-0.80)
	Emotional Symptoms	0.59 (0.47-0.69)
	Conduct Problems	0.66 (0.55-0.74)
	Hyperactivity/Inattention	0.68 (0.58-0.76)
	Peer Relationship Problems	0.58 (0.45-0.68)
	Prosocial Behavior	0.55 (0.43-0.66)

^a^All correlations were significant at the *P*<.001 level.

^b^Calculated for 137 participants because of missing data.

^c^Calculated for 133 participants because of missing data.

^d^Calculated for 134 participants because of missing data.

^e^Calculated for 139 participants because of missing data.

Intraclass correlation coefficients between 0.50 and 0.75 are considered moderate, whereas values above 0.75 are considered high [42]. The intraclass correlation coefficient between the web-based and paper formats of ASSQ was moderate and significant (*P*<.001). There were also moderate significant (*P*<.001) intraclass correlations found between the web-based and paper formats of the overall scale and subscales of the ADHD-RS and the DCDQ, and the P-SDQ subscales.

### Mean Differences Between the Web-Based and Paper-and-Pencil Formats

Table 4 shows the mean scores and standard deviations of both formats of the ASSQ, ADHD-RS, DCDQ, and P-SDQ.

**Table 4:** Mean scores for the web-based and paper formats of ASSQ, ADHD-RS, DCDQ, and P-SDQ (N=140).

Scale and subscales		Web-based, mean (SD)	Paper, mean (SD)	*t* test (*df*)	*P* value	Cohen *d*
Autism Spectrum Screening Questionnaire		4.10 (5.46)	3.99 (4.67)	0.31 (136)	.76	0.02
**Attention-Deficit/Hyperactivity Disorder Rating Scale**						
	Total	4.88 (7.08)	6.14 (6.94)	2.83 (132)	.005	0.18
	Inattention	2.64 (3.84)	3.22 (3.69)	2.15 (133)	.03	0.22
	Hyperactivity/Impulsivity	2.22 (3.46)	2.92 (3.62)	3.22 (136)	.002	0.20
**Developmental Coordination Disorder Questionnaire**						
	Total	58.85 (11.53)	57.74 (10.52)	1.55 (136)	.12	0.09
	Control During Movement	22.69 (4.89)	22.21 (4.48)	1.57 (136)	.12	0.06
	Fine Motor/Handwriting	16.54 (3.41)	16.36 (3.66)	0.73 (138)	.47	0.05
	General Coordination	19.72 (4.35)	19.26 (3.95)	1.61 (139)	.11	0.11
**Parent-rated Strength and Difficulties Questionnaire**						
	Total Difficulties	7.42 (4.83)	7.80 (4.72)	1.29 (139)	.20	0.08
	Emotional Symptoms	1.74 (1.69)	1.88 (1.76)	1.08 (139)	.28	0.07
	Conduct Problems	1.82 (1.50)	1.94 (1.51)	1.08 (139)	.28	0.08
	Hyperactivity/Inattention	2.67 (2.16)	2.76 (2.17)	0.63 (139)	.53	0.05
	Peer Relationship Problems	1.19 (1.37)	1.22 (1.36)	0.27 (139)	.79	0.02
	Prosocial Behavior	7.86 (1.92)	7.77 (2.12)	0.53 (139)	.60	0.05

There was no significant difference between the web-based and paper formats for the total mean score of the ASSQ (*P*=.76). Web-based scores were significantly lower than those of the paper format for the total mean scores of the ADHD-RS (*t*_132_=2.83, *P*=.005, Cohen *d*=0.18) and for its subscales (Inattention: *t*_133_=2.15, *P*=.03, Cohen *d*=0.22; Hyperactivity/Impulsivity: *t*_133_=3.21, *P*=.002, Cohen *d*=0.20). We found no significant differences between the web-based and paper formats for the mean scores on the DCDQ total (*P*=.12) and subscales (Control during Movement: *P*=.12; Fine Motor/Handwriting: *P*=.47; General Coordination: *P*=.11). Similarly, there were no significant differences between the web-based and paper formats for total P-SDQ scores (*P*=.20) and mean subscale scores (Emotional Symptoms: *P*=.28; Conduct Problems: *P*=.28; Hyperactivity/Inattention: *P*=.53; Peer Relationship Problems: *P*=.79; Prosocial Behavior: *P*=.60).

## Discussion

### Principal Findings

The purpose of this study was to examine the interformat reliability of the web-based versions of the ASSQ, ADHD-RS, DCDQ, and SDQ by comparing the internal consistency, intraclass correlation, and mean score differences of their web-based and paper formats.

For the ASSQ, ADHD-RS, and DCDQ, the McDonald ω coefficients were sufficient for both the web-based and paper versions, similar to findings in previous studies [19,22,38] that calculated Cronbach α. These results indicate that the web-based format of these questionnaires has good internal consistency. The McDonald ω coefficients for the Total Difficulties, Hyperactivity/Inattention, and Prosocial Behavior subscales of both the web-based and paper versions of the P-SDQ were also good. The McDonald ω coefficient for the paper version of the Emotional Symptoms subscale in this study was also good; however, it was unsatisfactory for the Conduct Problems and Peer Relationship Problems subscales of both the web-based and paper versions. The McDonald ω coefficient for the web-based version of the Emotional Symptoms subscale was also relatively low. These results are similar to those found in studies [25,39,43] that calculated Cronbach α for the paper format versions. Therefore, our findings suggest that, similar to the paper format, there are a few difficulties in using the web-based format of the P-SDQ for assessing externalizing and internalizing problems in children.

We found that there were significant (*P*<.001) moderate positive intraclass correlations between the web-based and paper formats of the ASSQ, ADHD-RS, DCDQ total scores, and Total Difficulties score of the P-SDQ. Similar to this study, several earlier studies [44-46] on the equivalence between web-based and paper formats of self-report questionnaires meant to assess psychiatric symptoms have reported moderate significant correlations, suggesting that web-based questionnaire administration was a reliable alternative to using the paper format. Hence, it seems that the questionnaires assessing neurodevelopmental disorders, such as the ASSQ, ADHD-RS, DCDQ, and P-SDQ, can be made available and administered in web-based situations as well. However, another study [27] had earlier pointed out that the agreement rate between the web-based and paper formats was higher for objective factual questions than for questions based on personal subjective evaluation. The questionnaires used in this study were subjective evaluations of children's developmental status by parents, which may have impacted the correlation values.

Furthermore, the analyses revealed that there were no significant differences in the ASSQ total mean scores between the web-based and paper formats (*P*=.76). This result suggests that the web-based version of the ASSQ is equal in quality to that of the paper version. However, there were significant differences in the ADHD-RS total and subscale mean scores between the web-based and paper formats (total: *P*=.005; Inattention: *P*=.03; Hyperactivity/Impulsivity: *P*=.002). Previous studies [33,36,47] have reported a significant difference in mean scores between web-based and paper formats; however, due to the small effect size (Cohen *d*=0.14-0.27), it was determined that the statistically significant difference in mean scores was not clinically meaningful in practice. In light of this finding, web-based questionnaires are a potential substitute for paper-based questionnaires. The effect sizes obtained in this study were also small (Cohen *d*=0.18-0.22). Furthermore, the web-based version McDonald ω values in this study were slightly higher than those of the paper format. These results suggest that the ADHD-RS is applicable for web-based utilization. We found that there were no significant differences in the DCDQ total and subscale mean scores between the web-based and paper formats (total: *P*=.12; Control during Movement: *P*=.12; Fine Motor/Handwriting: *P*=.47; General Coordination: *P*=.11). Additionally, we also confirmed that there were no significant differences in the P-SDQ total and subscale mean scores between the web-based and paper formats (total: *P*=.20; Emotional Symptoms: *P*=.28; Conduct Problems: *P*=.28; Hyperactivity/Inattention: *P*=.53; Peer Relationship Problems: *P*=.79; Prosocial Behavior: *P*=.60). These results suggest that both the DCDQ and P-SDQ web-based formats are equivalent in quality to those of the paper format.

### Strengths and Limitations

The evidence found in this study supports the applicability of the web-based versions of the ASSQ, ADHD-RS, DCDQ, and P-SDQ. It has been pointed out that children with possible neurodevelopmental disorders may be overlooked during developmental health check-ups in Japan [48], and there is a lack of specialized organizations capable of assessing neurodevelopmental disorders. Furthermore, in the current COVID-19 pandemic, it is also difficult to conduct in-person evaluations. This study, which shows the applicability of web-based questionnaires assessing neurodevelopmental disorders, has the potential to improve early detection and intervention for these disorders in regions where specialized services are lacking and under the present pandemic conditions.

However, there are some limitations to this research. First, the discriminant validity of the web-based version of each questionnaire was not confirmed in this study. This is because this study used a cross-sectional research design as part of a community developmental health check-up. Therefore, it is unclear whether the children included in this study had been diagnosed with neurodevelopmental disorders or experienced other emotional or behavioral problems or deficits that do not meet the criteria for a clinical diagnosis. It is necessary to verify the discriminant ability of the web-based version of each questionnaire for both clinical and nonclinical groups. Second, the raters who evaluated the children’s condition in this study were mostly parents. Previous research [17,49,50] conducted with paper-based questionnaires has examined the psychometric properties of the teacher-rated version of each questionnaire that was used in this study. In the future, we also need to clarify the psychometric properties of the teacher-rated web-based version of the questionnaires used in this study compared to the parent-rated web-based version. Third, we were not able to examine the impact of the order in which the questionnaires were administered. Previous studies [26,33] have confirmed that the order in which web- and paper-based questionnaires are administered has an effect on the scores of those questionnaires. In the future, it will be necessary to investigate the effect of the order of administration of the questionnaires in the research design. Fourth, the age of the children in this study was limited to 5 years. Previous studies [17,38,49,50] on the paper-based ASSQ, ADHD-RS, and SDQ have been conducted with school-age children. It is necessary to investigate the psychometric properties of the web-based version of the ASSQ, ADHD-RS, DCDQ, and P-SDQ among school-age children. Fifth, we did not control for the computer used by the participants in this study. We need to test whether the type of computer affects their responses in the future. Finally, this study was conducted in one medium-size city in Japan, thereby limiting the generalizability of its findings to other regions.

### Conclusions

This study examined the interformat reliability of the web-based versions of questionnaires for assessing neurodevelopmental disorders. Our findings showed that the web-based versions of the ASSQ, ADHD-RS, DCDQ, and P-SDQ had the same level of internal consistency, intrarater reliability, and equality as their paper versions. These results indicate the web applicability of these questionnaires for assessing neurodevelopmental disorders.

## Appendix

Multimedia Appendix 1 Participants who took part in the paper-and-pencil survey only (N=368).

## Abbreviations

ADHD-RS: Attention-Deficit/Hyperactivity Disorder Rating Scale
ASSQ: Autism Spectrum Screening Questionnaire
DCDQ: Developmental Coordination Disorder Questionnaire 2007
DSM-5: Diagnostic and Statistical Manual for Mental Disorders (Fifth Edition)
P-SDQ: Parent-reported Strengths and Difficulties Questionnaire
SDQ: Strengths and Difficulties Questionnaire
