# Supplementary material for: Interformat Reliability of Web-Based Parent-Rated Questionnaires for Assessing Neurodevelopmental Disorders Among Preschoolers: Cross-sectional Community Study
Source: JMIR Pediatr Parent. 2021 Feb 4;4(1):e20172. doi: 10.2196/20172 (PMC8078684; doi:10.2196/20172)
Supplement: Multimedia Appendix 1 [file pediatrics_v4i1e20172_app1.docx]

| Characteristics | | Value (N=368)^a^, n (%) |
| --- | --- | --- |
| **Children’s gender** | |  |
|  | Boy | 200 (54.3) |
|  | Girl | 168 (45.7) |
| **Children’s age (months)** | |  |
|  | 57 months | 62 (16.8) |
|  | 58 months | 69 (18.8) |
|  | 59 months | 59 (16.0) |
|  | 60 months | 70 (19.0) |
|  | 61 months | 52 (14.1) |
|  | 62 months | 56 (15.2) |
| **Respondent** | |  |
|  | Mother | 352 (95.7) |
|  | Father | 13 (3.5) |
|  | Unknown | 3 (0.8) |
| **Childcare during daytime**^b^ | |  |
|  | Nursery school | 287 (80.4) |
|  | Kindergarten | 66 (18.5) |
|  | Mother | 3 (1.0) |
|  | Grandmother | 1 (0.3) |
| **Household income**^c^ **(JPY**^d^**)** | |  |
|  | <2 million | 28 (7.8) |
|  | 2-4 million | 99 (27.4) |
|  | 4-7 million | 143 (39.6) |
|  | 7-10 million | 46 (12.7) |
|  | >10 million | 24 (7.2) |
|  | Don’t know | 21 (5.8) |

^a^This data was collected 3 months prior to the web-based survey (June 2018).

^b^calculated for 357 participants because of missing data.

^c^calculated for 361 participants because of missing data.

^d^JPY: Japanese Yen; an approximate exchange rate of US $1= 103.80 JPY.
